# Catastrophic health expenditure and associated factors among households of non community based health insurance districts, Ilubabor zone, Oromia regional state, southwest Ethiopia

**DOI:** 10.1186/s12939-023-01847-0

**Published:** 2023-03-09

**Authors:** Nigusu Getachew, Hailu Shigut, Gebeyehu Jeldu Edessa, Elias Ali Yesuf

**Affiliations:** Department of Health Policy and Management, Faculty of Public Health, Institute of Health, Jimma University, P.O. Box 378, Jimma, Ethiopia

**Keywords:** Out of pocket payment, Catastrophic health expenditure, Impoverishment, And Ilubabore zone

## Abstract

**Background:**

Out-of-pocket health expenditure is the proportion of total health expenditure that is paid by individuals and households at the time of health service. Hence, the objective of this study is to assess the incidence and intensity of catastrophic health expenditure and associated factors among households in non-community-based health insurance districts in the Ilubabor zone, Oromia National Regional State, Ethiopia.

**Method:**

A community-based cross-sectional study design was employed in the Ilubabor zone on non-community-based health insurance scheme districts from August 13 to September 2, 2020, and 633 households participated in the study. A multistage one cluster sampling method was used to select three districts out of seven districts. Data was collected by using a structured mix of open and close-ended pre -tested questionnaires by face-to-face interviewing. A micro-costing/bottom up approach was used for all household expenditure. After checking its completeness, all household consumption expenditure was done by mathematical analysis using Microsoft Excel. Binary and multiple logistic were done using 95%CI and significance was declared at *P* < 0.05.

**Results:**

The number of households that participated in the study was 633, with a response rate of 99.7%. Out of 633 households surveyed, 110 (17.4%) were in catastrophe, which exceeds 10% of total household expenditure. After medical care expenses, about 5% of the households moved downward from the middle poverty line to extreme poverty. Out-of-pocket payment AOR: 31.201: 95% CI (12.965–49.673), daily income less than 1.90 USD AOR: 2.081: 95% CI (1.010–3.670), living a medium distance from a health facility AOR: 6.219: 95% CI (1.632–15.418), and chronic disease AOR: 5.647: 95% CI (1.764–18.075.

**Conclusion:**

In this study, family size, average daily income, out of pocket payment and chronic diseases were statistically significant and independent predictors for household catastrophic health expenditure. Therefore, to overcome financial risk, the Federal Ministry of Health should develop different guidelines and modalities by considering household per capita and income to improve the enrolment of community-based health insurance. Also, the regional health bureau should improve their budget share of 10% to increase the coverage of poor households. Strengthening financial risk protection mechanisms, such as community-based health insurance, could help to improve healthcare equity and quality.

## Introduction

Catastrophic health spending is defined as out-of-pocket spending exceeding 10% of total consumption or income by the budget-share approach with two thresholds, as well as out-of-pocket spending exceeding 40% of nonfood consumption. Impoverishment is defined as occurring when a household’s consumption, including out-of-pocket spending, is higher than the poverty line but its consumption, excluding out-of-pocket spending, is lower than the poverty line. The idea is that a household that is impoverished by out-of-pocket spending was forced by an adverse health event to divert spending away from non-medical budget items such as food, shelter, clothing, etc. to such an extent that its spending on these items is reduced below the level indicated by the poverty line. Impoverishment can be computed as the change in poverty headcount with and without out-of-pocket spending included in consumption or income [1] .Out-of-pocket spending surpassing 10% of total consumption or income by budget share approach with two thresholds, as well as out-of-pocket spending exceeding 40% of nonfood consumption, is considered catastrophic health spending. When a household’s consumption, including out-of-pocket spending, exceeds the poverty level but its consumption, excluding out-of-pocket spending, falls below the poverty line, it is said to be impoverished. The concept is that a household that has become impoverished due to out-of-pocket spending has been compelled to redirect spending away from non-medical budget items such as food, shelter, clothes, and other necessities to the point that spending on these things falls below the poverty line. Anticipating the type of illness and protecting the society at risk from excessive healthcare spending that can lead to poverty requires structured healthcare policies. The goal should be to minimize inequality by improving access to health care and establishing a pre-payment system to avoid financial disaster. The core concept of health funding is equity. It is founded on national solidarity and shared responsibility, with the healthy and wealthy sharing the financial burden in order for the sick and destitute to receive treatment [2].

Globally, 11.7% of the world’s population was predicted to have catastrophic health spending, defined as out-of-pocket expenses surpassing 10% of household total consumption or income. The results are 179 million, or 2.6%, at the 25% threshold. Persons in Latin America and Asia have the highest rates of people who spend more than 10% or 25% of their household’s entire consumption or income out of pocket. Latin America and the Caribbean had the highest 10-percentage-point rate (14.8%). Asia has the second-highest percentage (12.8%), and it is in this region where the majority of persons facing catastrophic payments live. Ninety-seven million individuals, or 1.4% of the world’s population, are poor due to out-of-pocket spending at the 2011 PPP $1.90 per day poverty threshold [1].

The very best use of health care services was in Namibia, at 18% for inpatient care and 41% for outpatient care, and the lowest was in the Democratic Republic of the Congo (DRC), at 4% for inpatient care and seven for outpatient care. Average out-of-pocket spending for health care was highest in Liberia and lowest in Rwanda. All told, insurance coverage stands out as a crucial factor affecting the magnitude of out-of-pocket (OOP) health expenditure across four countries, but the results are mixed. In the DRC and Rwanda, insurance coverage was related to lower out-of-pocket expenditures for both inpatient and outpatient care services, while in Liberia and Namibia, it was definitely related to higher out-of-pocket expenditures [3].

Overall health care in Ethiopia is underfinanced both in absolute terms and in comparison to sub-Saharan African (SSA) standards. As an example, the per-capita national health expenditure for the country was reported to be $20.77 during the year 2011, while the SSA average was $93.55. This per capita health expenditure for Ethiopia is additionally well below the WHO’s recommended US $30–40 per person needed to cover essential health care in low-income countries [4].

Ethiopian health policy is committed to facilitating access to services by ensuring financial risk protection [5]. Financial protection through community-based insurance (CBHI) is the one engine for the implementation of the health sector transformation plan [6].

Emphasizing complete access to and coverage of health services, the sustainable development target focuses on financial risk protection for universal health coverage. Many countries have weak health financing systems that lead families to out-of-pocket payments and financial catastrophe or impoverishment at the time of illness [7].

In Ethiopia, to enhance access to health care and reduce the burden of OOP expenditure, community-based health insurance started in June, 2011. To start out the CBHI service, about 60% of the households should be enrolled, and district and regional governments are expected to hide the cost of providing a fee waiver to the poorest 10% of the population, approximately called “indigent groups.” Concerning the significance of CBHI, findings revealed that the risk of being… impoverished by OOP health expenditure is 7% for CBHI members and 19% for non-members at the 15% threshold, and 3% for members and 9% for non-members at the 25% threshold [8, 9]. Community-based insurance was started in 50% of the Ilubabor districts, but enrollment is incredibly low. It is imperative to know the catastrophic health expenditure, according to my knowledge and access until the writing of this research, there is no research conducted directly on the issues of in the study setting that helps to know the out of pocket expenditure. Accurate knowledge about impoverishments and catastrophic health expenditure is essential and helps us to formulate and prioritize health care policies and interventions and eventually to allocate health care resources in accordance with budget constraints in order to achieve policy efficiently. Therefore, during this study, the incidence and intensity of catastrophic health expenditure and factors related to CHE were analyzed.

## Methods

### Study setting and period

The study was conducted in three randomly selected non-CBHI districts of the Ilubabore Zone (Ale, Becho, and Didu), southwest of Ethiopia, from August 13 to September 2, 2020. The zone has a total population of 9, 33,325 with a total of 194,443 houses and out of 14 districts, 7 districts, and 20.2% (39239) of them are not included in the CBHI. The zone has one specialized hospital, one general hospital, one urban health center, 38 rural health centers, and 273 functional health posts out of 23 urban and 263 rural kebeles [10].

### Study design and participant

A community-based cross sectional study design was employed. All and randomly selected households living in non-CBHI districts of the Ilubabor zone were the source and study population, respectively. All households in non-CBHI scheme districts and households that have stayed for more than 1 year were included, and members of private insurance were excluded from the study.

### Sample size determination and sampling procedure

Due to a lack of such studies, a single population formula was used; assuming 95% confidence interval and 50% prevalence (P), and a precision of 5% was taken to account for the sampling variability of multi-stage sampling. A design effect of 1.5 was used to account for the sampling variability of multi-stage sampling, requiring a total of 634 study participants. A multi-stage sampling technique was employed, taking the districts as primary sampling units (PSU), the kebeles from the selected districts as secondary sampling units (SSU), and the households from the selected kebeles as tertiary sampling units (TSU). Thirty percent of the districts and Kebeles were chosen through a lottery system. The sample size was proportionally allocated to the selected kebeles. Finally, a simple random sampling (SRS) method was used to obtain the interviewee for an interview at a household (HH) level. Households those who can fulfill the inclusion criteria was listed by their household number from master family index (MFI) of family folders by using community health information system (CHIS) and household’s numbers was obtained and used as sampling frame.

### Data collection instrument

Data was collected by the use of open and close-ended questionnaires by face-to-face interviewing of respondents from the household. It was adapted after reviewing different literature [5, 11, 12]. Data collection tools were prepared in English and then translated into the language (Amharic and Afaan Oromo), i.e., by a language expert, and then back to English by another language expert to ensure consistency. Six BSc Nurse Data collectors who were fluent in speaking and writing the local language participated in the data collection process, using a pretested interviewer-administered structured questionnaire for the quantitative part. To ensure data quality, a pretest was conducted on 10% of the sample size to determine the clarity of the items and the consistency of the responses. During actual data collection, the filled questionnaires were checked for consistency and completeness each day and submitted to the supervisors.

#### Study variable

Dependent variable was catastrophic health expenditure and independent variables were Socio-demographic and socio economic variables, Environmental factors, Coping mechanisms and Health status.

### Operational definitions

Ability to pay: if income remains after spending on basic subsistence needs and if the household’ income remains above the poverty line after spending on health services.

Catastrophic health expenditure occurs when out-of-pocket health-care costs exceed 10% of total household spending.

Intensity of catastrophic health expenditure: prevalence of catastrophic health expenditure at threshold above 20% of total household expenditure.

Consumption expenditure: a preferred measure of living standards, particularly food consumption, accounts for a large proportion of household expenditure, and the limited consumption of non-food is likely to be sensitive to household size and access to cash.

Impoverishment:-household that is impoverished by out-of-pocket spending and was forced by an adverse health event to divert spending away from non-medical budget items such as food, shelter, clothing, etc. In another way, households’ declined from the upper quartile range to the lower and the lower one to below the poverty line.

Total household expenditure evaluation techniques: - Different techniques for estimating the cost of total household expenditures were used. Individual household expenditures, healthcare spending, and individual level data on outpatient attendance (over a six-month recall period) and inpatient hospitalization (over a 12-month recall period) were collected and computed as follows. By using total household expenditure, then it was calculated as follows. To determine the cut point of catastrophic health expenditure, there were two assumptions: if health expenditure exceeds 40% of nonfood expenditure OR if health expenditure exceeds 10% of total household expenditure, in this article, total expenditure was used. If the household catastrophe exceeds 10% of total household expenditure,

$$\textrm{CHE}=\textrm{OOPHE}/\textrm{THE}=\textrm{Total}\ \textrm{household}\ \textrm{expenditure}$$

$$\textrm{THE}>10\%\textrm{and}\ \textrm{not}\ \textrm{if}\ \textrm{OOPHE}/\textrm{THE}<10\%,\textrm{OOPHE}=\textrm{Out}\ \textrm{of}\ \textrm{pocket}\ \textrm{health}\ \textrm{expenditure}$$

$${\displaystyle \begin{array}{c}\textrm{Yes}=1,\textrm{OOPHE}/\textrm{THE}>10\%\\ {}\textrm{No}=2,\textrm{OOPHE}/\textrm{THE}<10\%\end{array}}$$

### Data quality control

The questionnaire was developed and pre tested at similar district which is not included in CBHI scheme and was translated to the language Afan Oromo and Amharic. Two days training was given and also during data collection data collectors writes unique number on the upper side of the households’ door and the senior supervisor check 20% of study unit by using rapid convenience survey (RCSM). For any missing or inconsistent data, it was returned back to the respective data collector for re-interview and also re-visit was done to those households who were not available on the first day of interview. The principal investigator prepared the template and entered data using Epi Data version 3.1 then exported to SPSS version 23.0. Frequencies were used to check for missed values and outliers. Any error identified at this time was corrected after revision of the original data using the code numbers.

### Data processing and analysis

The collected data were checked for consistency, and then coded. CHE to households associated with health service OOP expenses was calculated by computing OOP expenditure incurred minus any reimbursements from third-party payers divided by annual households’ total expenditure. Also one-way sensitivity analysis was done to check, variation done at 15, 10 and 5% threshold and the finding was varied 13.2, 17.4 and 23.4% respectively. At the lower thresholds of 5, 10 and 15%, the likelihood of CHE is higher. Cost analysis was done by using Microsoft excel and data was entered to Epi Data version 3.1 then exported to SPSS version 23.0 analysis, binary logistic regression analysis was used to describe association of independent variables with CHE. For the descriptive statistical part, means, proportions, tables, graphs, and charts were used to summarize and present the results of the study. Variables with a *p*-value ≤0.25 on simple logistic regression were taken as candidates for multivariable logistic regression and factors predicting outcome variable were identified using at a significance level of p-value < 0.05. The goodness of fit was checked with the Hosmer–Lemeshow test (*p* = 0.35). Using an adjusted odds ratio (AOR) with 95% confidence interval, the associations of dependent and independent variables were interpreted. Data were entered into epi-Data version 3.1 by two persons separately and the two files were validated for consistency, and necessary corrections were made before exporting the data into SPSS for analysis.

## Result

### Characteristics of study participants

Out of 634 households, 633 (99.7%) responded to the questionnaires, making the response rate 99.7%. The majority of the participants were males (547, or 86.4%). The majority of the participants were 185 (29.2%) aged between 20 and 29 years. More than two-thirds of the participants were farmers (490, or 77.4%) and almost half of the participants (314, or 49.6%) had completed primary education (See Table 1).

**Table 1:** Frequency distribution of socio-demographic characteristics of the respondents for the study of catastrophic health expenditure among households of non CBH districts, Ilubabor zone, southwest, Ethiopia, 2020 (*n* = 633)

Variables		Catastrophic			
		Yes	No	Total	%
Age	20–29	23	162	185	29.3
	30–39	20	150	170	26.8
	40–49	33	122	155	24.5
	50–59	16	50	66	10.4
	60–69	12	36	48	7.6
	Above 70	2	7	9	1.4
Sex	Male	92	455	547	86.4
	Female	18	68	86	13.6
Marital status	Single	7	24	31	4.9
	Married	91	458	549	86.7
	Divorced	2	11	13	2
	Widowed	11	29	40	6.4
Educational status	Can’t read and write	17	105	122	19.6
	Only read and write	15	44	59	9.6
	Primary education	49	265	314	49.8
	Secondary education	24	56	70	11
	Above secondary	13	50	63	10
Presence of < 5 children	Yes	46	218	264	42
	No	65	304	369	58
Pregnant Women	Yes	2	38	40	6.3
	No	108	485	593	93.7
Presence of Age > 65 yrs	Yes	9	44	53	8.4
	No	101	479	580	91.6
Occupational status of the household head	Manager	2	2	4	.6
	Professionals GO.	4	29	33	5.3
	Professionals NGO	2	9	11	1.8
	Technicians associated	8	9	17	2.4
	Service and sales worker	15	45	60	9.6
	Farmer	77	413	490	77.4
	Others*	3	15	18	2.9
Place of residence	Rural	43	224	267	80
	Urban	15	51	66	20
Family size	< 5	14	315	329	52
	> 5	97	207	304	48
Average household daily income USD	<1.90	52	89	141	22.3
	1.91–3.20	36	276	312	49.3
	3.21–5.5	21	112	133	21
	> 5.5	2	45	47	7.4

*USD* United States Dollar

### Catastrophic health expenditure and coping mechanisms

#### Health status and health service utilization of the respondents

Out of 633 households surveyed, 395 (62.4%) of the households had a history of illness in the past year during the study period. 179 (45.2%) of household family members received medical care above the threshold of 10% of total household consumption, and 110 (17.4%) of the households incur catastrophic health expenditure. And when the intensity of catastrophic health expenditure was computed, 24 (7%) of the households incurred catastrophic health expenditure (See Table 2).

**Table 2:** Health status and the reason why not seeking medical care 2020

Variables		Frequency	Percent
Member of family encountered any illness	Yes	395	62.4
	No	238	37.6
	Total	633	100.0
Member of family seek any medical care	Yes	179	45.2
	No	117	54.8
	Total	396	100.0
The reason of household no going to any medical care	Visiting health post only	57	26
	Using home remedy	18	9
	Perceiving the illness is not severe and buying anti pain from pharmacy	39	18
	Because financial barrier	94	43
	Using traditional healers	9	4
	Total	217	100
Catastrophic health expenditure at 10% of total household expenditure	Yes	110	17.4
	No	523	82.6
	Total	633	100

Concerning households’ coping mechanisms in response to health expenditure, 59.6% of the households using direct out of pocket at the time of service, followed by pawning their assets, extra working, borrowing from others, and drawing money from their savings, pensions, and re-imbursement at the time of service were 27.7, 26.6, 20, 15, 13, and 9% respectively to overcome the burden of out of pocket payment.

Out of the total households surveyed, 11.1% of those receiving health care were in moderate poverty, while 8.4 and 6.6% of those receiving health care were in extreme poverty and above the poverty line, respectively. After health service expenditure, 13.5% of the households were in extreme poverty; 10.5% of them were in middle income.

#### Annual household income and expenditure by USD

**Fig. 1:**
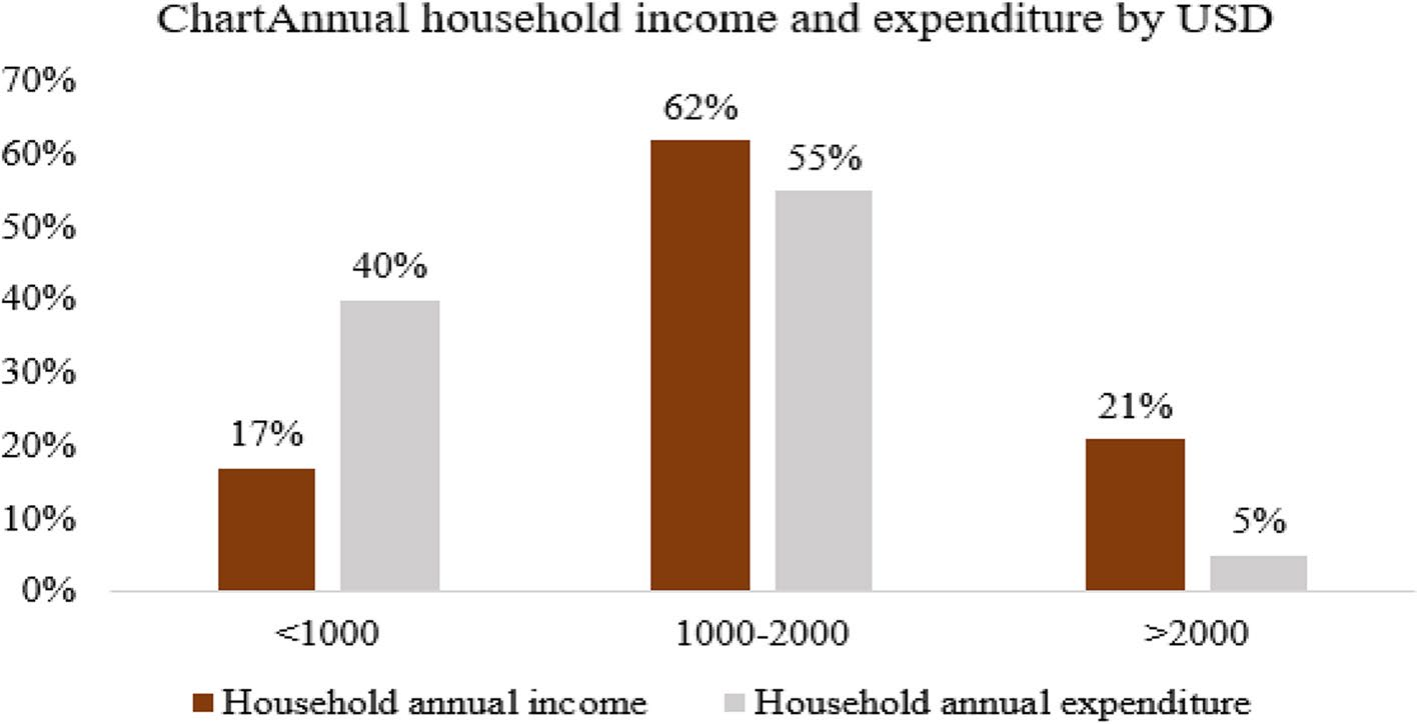
The households’ average annual income and total expenditure distribution by USD of the respondents for the study of catastrophic health expenditure and associated factors among households of non CBH districts, Ilubabor zone 2020

**Fig. 2:**
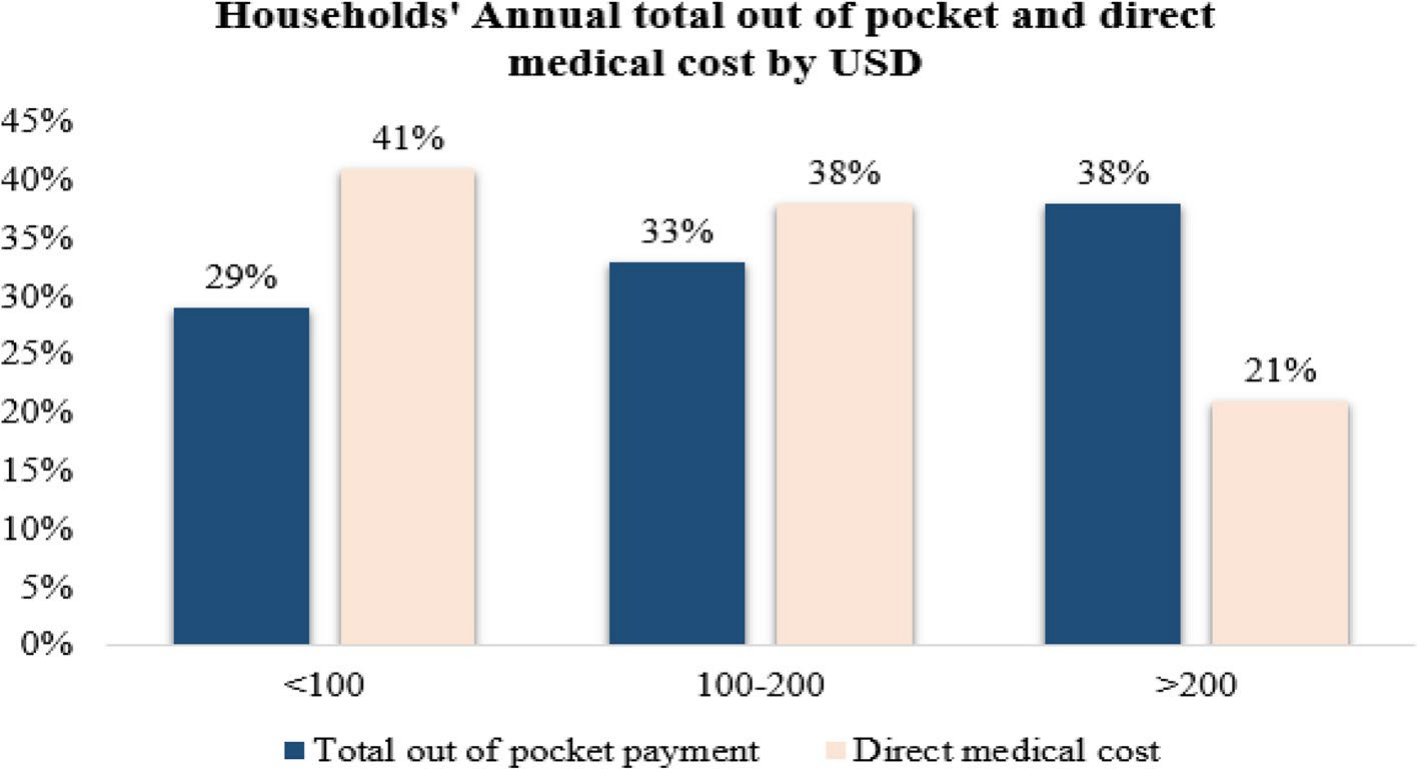
Distribution of average annual total out of pocket health expenditure and direct medical cost by USD for the study of catastrophic health expenditure and associated factors among households of non CBH districts, Ilubabor zone, 2020

**Fig. 3:**
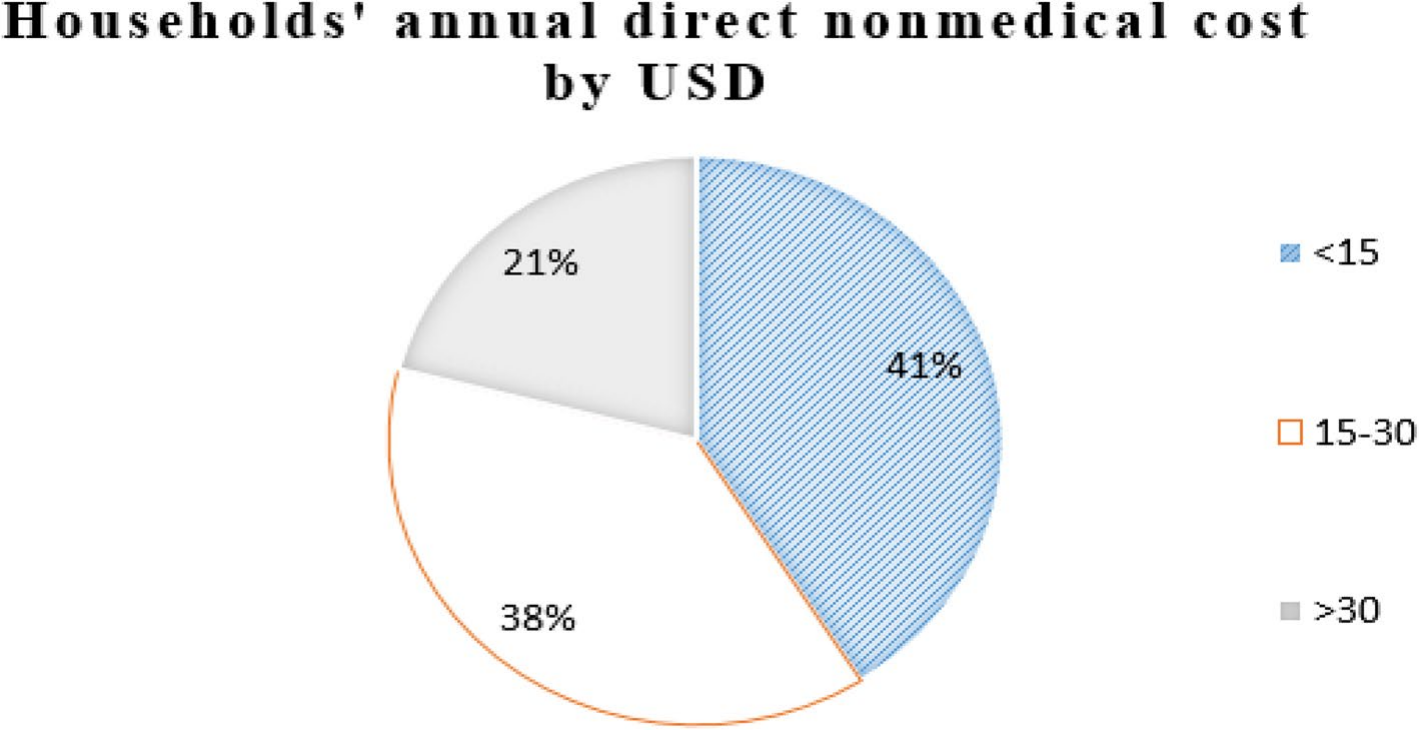
Distribution of direct nonmedical cost for the study of catastrophic health expenditure and associated factors among households of non CBH districts, Ilubabor zone, 2020

**Fig. 4:**
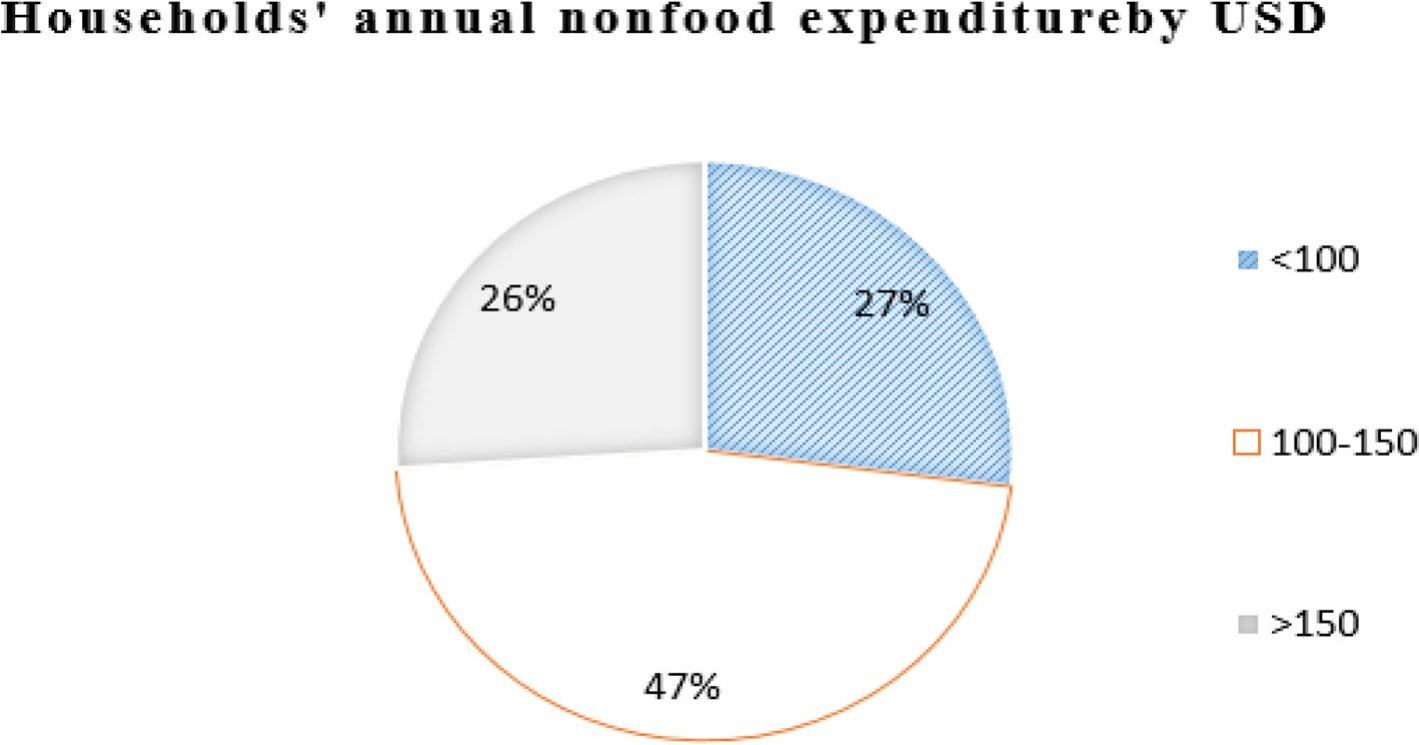
Distribution of household annual non-food expenditure by USD for the study of catastrophic health expenditure and associated factors among households of non CBH districts, Ilubabor zone, 2020

**Fig. 5:**
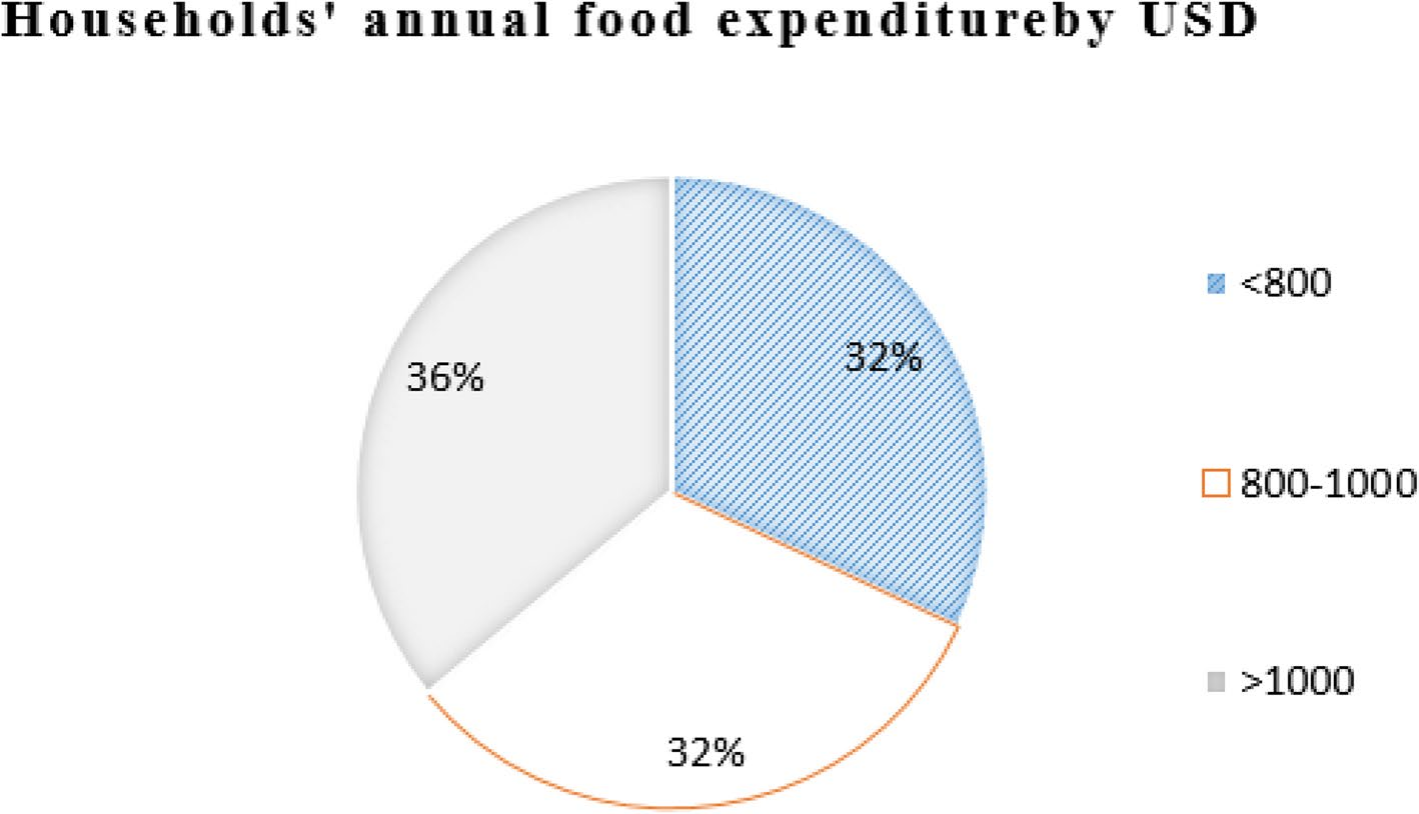
Distribution of out of pocket share of household annual food expenditure by USD for the study of catastrophic health expenditure and associated factors among households of non CBH districts, Ilubabor zone 2020

Out of the total households surveyed, 392 (62%) of the households’ average annual income was between 1000 and 2000 USD and 21% of them were above 2000 USD. When their average annual consumption was computed, 55% of the households expended between 1000 and 2000 USD and 40% of the households’ consumption was below 1000 USD. Regarding the annual health service expenditure computed as the summation of direct medical and nonmedical costs, 38% of the household total health expenditure was above 200 USD, and 33% of the household spent on health between 100 and 200 USD. When medical costs were computed, 41% of the households expended on direct medical costs were below 100 USD and 38% of the households between 100 and 200 USD. Out of total non-medical costs, 40% of the households spent on direct non-medical costs were below 15 USD and 31% of the households spent above 30 USD. Regarding nonfood expenditure, the majority, or 47%, of the households expended between 100 and 150 USD and 27% of them below 100 USD. Also, when annual food expenditure was computed, 36% of the household spent on food above 1000 USD and 32% of the household expended below 800 USD annually (Figs. 1, 2, 3, 4 and 5).

### Factors associated with catastrophic health expenditure

In bi-variable logistic regression, from variables under socio demographic, environmental factors, health service utilization types and health facility, coping mechanism and health related variables associated with catastrophic health expenditures were age of household head, household occupation, family size, presence of pregnant mother, place of residence, distance from health facility, average daily income, health service at private facility, transportation by foot, ambulance, Bajaj, car and minibus, payment made from saving and pension, out of pocket payment, pawn of asset for medical expenses, inpatient service and presence of chronic disease were significant at *P* < 0.25 and selected as candidate for multi-variable logistic regression. Finally, family size, average daily income, distance from health facility, presence of chronic disease, out of pocket payment and transportation by Ambulance service were significantly associated with catastrophic health expenditure at *P* < =0.05 (see Table 3).

**Table 3:** Multivariable logistic regression of socio-demographic, environmental factors and coping mechanisms of households for the study of catastrophic health expenditure and associated factors in Ilubabor Zone, 2020

Variables		COR,CI 95%	AOR,CI 95%	*P*-Value
Family size	<=5	1	1	
	> 5	11.096 (9.857–18.348)	10.069 8.290–20.307	0.001*
Average daily income USD	<1.90	2.073 (1.009–3.567)	2.081 1.010–3.670	0.020*
	1.91–3.20	0.318 (0.041–2.487)	0.387 0.047–3.183	0.377
	3.21–5.5	0.223 (0.027–1.828)	0.220 0.025–1.912	0.17
	> 5.5	1	1	
Distance from health facility	Nearest	1	1	
	Medium	4.008 5.797–18.941	6.2191.632–15.418	0.016*
	Distant and hard to reach area	1.609 0.846–3.061	4.538 0.493–8.773	0.182
Ambulance service	Yes	1	1	
	No	0.086 0.022–0.328	0.007 0.001–0.291	0.009
Out of pocket	Yes	22.556 7.509–41.032	31.20112.965–49.673	0.001**
	No			
Chronic disease	Yes	6.040 2.004–23.370	5.647 1.764–18.075	0.004
	N0	1	1	

*USD* United States Dollar

## Discussion

At a threshold of 10% total health expenditure, the incidence of catastrophic health expenditure in this study is 17.4%, which is greater than studies done in Kenya (6.57%), Palestine (6.7%), and Jordan (2.7%) and is nearest to Egypt, where 20% of households incur catastrophic health expenditure. Lower middle-income households were affected by 7% more than upper middle-income households [11, 13, 14].

This might be due to the economic variation and different characteristics of the health system between the countries. The households’ average daily income has an inverse relationship with catastrophic health expenditure. The AOR of those households who had an average daily income of less than 1.90 USD was 2.081: 95% CI (1.010–3.670) *P* < 0.020 when compared with those households with lower middle income (1.91 and 3.20 USD) incurring catastrophic health expenditure. Also, comparative findings were observed with studies done in Kenya, Egypt, and Bangladesh [11, 14, 15]. This might be due to the probability of increment of total households’ expenditure as average daily income is increased, such as buying extra assets during the period of the study.

Households who were living a medium distance had an AOR of 6.219 at 95%CI: 1.632–15.418, *P* < 0.016) when compared to those who were leaving nearest to a health facility by increasing transportation costs, which is indicated by household members using ambulance service for transportation less likely affected by catastrophic health expenditure. AOR = 0.007; 95% CI (0.001–0.291), *p* < .009. Finding was observed in a study done in Kenya, which shows transportation costs have a significant association with catastrophic health expenditure [11]. This might be due to patients traveling a long distance to visit health facilities where they incur additional transportation and food costs.

The AOR of households with a large family size is 10.069, at 95% CI (8.290–20.307, *P* < 0.001) when compared with less than five family members. This result is similar to a study done in Kenya, but the opposite finding was observed from a study done in Egypt [11, 13]. This is probably due to large family size. If total household expenditure had not increased with family size and the healthcare cost had increased because of large family size, there may be an increased chance of CHE. But a study in Egypt found that large family size is less likely to be affected by catastrophic health expenditure because large households take advantage of economies of scale in household consumption.

The AOR of households that were using out of pocket payment for health service during the time of service was 31.201, at 95% CI: 12.965–49.673: *P* < 0.001) when compared to those households not using out of pocket payment during health service. Comparative findings were also observed with studies done in Kenya and Egypt [11, 13]. The AOR of households with chronic disease is 5.647 at 95% CI (1.764–18.075: *P* < 0.004) when compared with households without chronic disease. This finding was similar to studies done in Kenya, Egypt, and Bangladesh which showed family members of households who had chronic diseases were more likely to be affected by catastrophic health expenditure than households without chronic diseases [11–13]. The AOR of households with chronic disease is 5.647 at 95% CI (1.764–18.075: P < 0.004) when compared with households without chronic disease. This finding was similar to studies done in Kenya, Egypt, and Bangladesh which showed family members of households who had chronic diseases were more likely to be affected by catastrophic health expenditure than households without chronic diseases [11–13]. This is probably because the presence of chronic disease in the family increases the consumption of health expenditure and may have the chance of increasing out-of-pocket health expenditure.

### Limitation of the study

Due to the duration of the study there may be recall bias on households’ total expenditure. So, to minimize recall bias, bottom up costing and 2 month history of illness and 1 year period history of hospitalization was used for analysis. During costing the study consider only costs associated with direct medical and nonmedical cost. Provider side cost and indirect cost related to any health service, wage loss, productivity loss due to chronic disease, disability or death not considered.

## Conclusion

The result the incidence of catastrophic health expenditure was 17.4% at cut point of 10% total household expenditure and 5% of households were impoverished due to medical expenses and the intensity of catastrophic health expenditure. Family size, average daily income, distance from health facility, presence of chronic disease, out of pocket payment and transportation by Ambulance service were statistically significant and independent predictors households catastrophic health expenditure due to out of pocket payments. All households in different income level will be affected by catastrophic health expenditure and impoverished. Households above poverty line can borrow money, sell assets or pawn assets for other households to get health service. But households living below poverty line highly affected by health service expenses and they don’t have more option to sell assets or pawn assets the final option is decreasing their food expenditure. Therefore, to overcome financial risk, all concerned bodies develop different guidelines and modalities by considering the household per capita income to improve the enrolment of community based health insurance. Minister of health should provide a legal basis for networking of CBHI schemes to create larger risk pools for the purposes of reinsurance and future integration of CBHI into SHI as a vehicle for UHC.

## Data Availability

The datasets generated and/or analyzed during the current study are not publicly available due to the agreement that we have with the sponsored institution not to share the data for third part and there are some irremovable identifier data but are available from the corresponding author on reasonable request.
